# Assessing health program performance in low- and middle-income countries: building a feasible, credible, and comprehensive framework

**DOI:** 10.1186/s12992-015-0137-5

**Published:** 2015-12-21

**Authors:** Onil Bhattacharyya, Kathryn Mossman, John Ginther, Leigh Hayden, Raman Sohal, Jieun Cha, Ameya Bopardikar, John A. MacDonald, Himanshu Parikh, Ilan Shahin, Anita McGahan, Will Mitchell

**Affiliations:** Women’s College Hospital, 76 Grenville St, Toronto, ON M5S 1B1 Canada; Department of Family and Community Medicine, University of Toronto, 500 University Avenue, Toronto, ON M5G 1V7 Canada; Rotman School of Management, University of Toronto, 105 St. George Street, Toronto, ON M5S 3E6 Canada; Toronto Health Organization Performance Evaluation (T-HOPE), University of Toronto, 105 St. George Street, Toronto, ON M5S 3E6 Canada; The Scarborough Hospital, 3030 Lawrence Avenue East, Suite 108, Toronto, ON M1P 2V5 Canada; Institute of Health Policy, Management and Evaluation, University of Toronto, 155 College Street, 4th Floor, Toronto, ON M5T 3M6 Canada; Department of Surgery, University of British Columbia, 950 West 10th. Avenue, Vancouver, BC V5Z 1 M9 Canada; Department of Anesthesia, University of Toronto, 123 Edward Street, Toronto, ON M5G 1E2 Canada; MIT Sloan School of Management, 30 Memorial Dr., Cambridge, MA 02142 USA

**Keywords:** Performance measurement, Innovation, Framework, Health service delivery, Low- and middle-income countries, Private sector

## Abstract

**Background:**

Many health service delivery models are adapting health services to meet rising demand and evolving health burdens in low- and middle-income countries. While innovative private sector models provide potential benefits to health care delivery, the evidence base on the characteristics and impact of such approaches is limited. We have developed a performance measurement framework that provides credible (relevant aspects of performance), feasible (available data), and comparable (across different organizations) metrics that can be obtained for private health services organizations that operate in resource-constrained settings.

**Methods:**

We synthesized existing frameworks to define credible measures. We then examined a purposive sample of 80 health organizations from the Center for Health Market Innovations (CHMI) database (healthmarketinnovations.org) to identify what the organizations reported about their programs (to determine feasibility of measurement) and what elements could be compared across the sample.

**Results:**

The resulting measurement framework includes fourteen subgroups within three categories of health status, health access, and operations/delivery.

**Conclusions:**

The emphasis on credible, feasible, and comparable measures in the framework can assist funders, program managers, and researchers to support, manage, and evaluate the most promising strategies to improve access to effective health services. Although some of the criteria that the literature views as important – particularly population coverage, pro-poor targeting, and health outcomes – are less frequently reported, the overall comparison provides useful insights.

## Background

Adapting health services to meet the rising demand and evolving health burden in low- and middle income-countries (LMICs) is key to improving health outcomes. Interest in the potential for health innovations to improve quality and access of health care for LMIC populations is growing rapidly [1–3]. Many organizations, including private providers, governments, donors, and social impact investors, have developed and supported innovative approaches to health services delivery for the poor. In particular, the private health sector, which includes for-profit and not-for-profit, formal and non-formal entities [4], plays a significant innovative role in influencing health policy and providing health care and supplies in LMICs [5, 6]. However, our evidence on what works, particularly in the private health sector of developing regions, is relatively weak [7], and greater understanding of the effectiveness, scale, and scope of private sector initiatives is needed [8]. Innovative programs are seldom evaluated in a way that allows for meaningful comparisons [9–11], and in rapidly changing health markets, formal evaluations are often too time consuming and costly for new interventions or rapidly evolving organizations [7]. We need new approaches to improve the knowledge base on health markets in LMICs, which is crucial for improving health policy and practice [12]. This requires a cohesive set of measures that balance credibility (relevant aspects of performance), comparability (across different organizations), and feasibility (available data).

Performance measurement frameworks seek to determine the activities and success of a program’s strategy and provide insights for future improvements [13]. Multiple performance frameworks have been designed to assess health systems [14, 15], health service delivery organizations, [13, 16] and health quality [17, 18]. Additional frameworks measure the impact of socially responsible businesses and social enterprises [19, 20].

While some of the performance measures in existing frameworks have been rigorously tested to determine their credibility, they face substantial challenges in comparability and feasibility. Measures face comparability challenges because they are often specific tocertain practices and health areas, making them difficult to apply across health areas and models. They face feasibility challenges because they often do not consider whether programs have the capacity to collect and report the necessary data, imposing burdens that may detract from service delivery, particularly for smaller and newer health programs.

Performance measures are relevant for multiple stakeholders. Funders and researchers must compare health programs to determine what activities they are undertaking and which are performing well. Program managers are interested in the minimum data set that is relevant to operations and to assess their performance relative to their peers. Meeting the goals of these stakeholders requires that performance measures are credible in assessing relevant aspects of performance, comparable in evaluating programs across different health areas and models, and feasible for programs. The measures need to achieve a balance among these three elements of assessment.

This paper presents a balanced framework for assessing the performance of health care programs in LMICs and elsewhere. This framework integrates important existing approaches and supplements them with novel operational criteria. The result is a template that organizations can use for reporting purposes and may also serve as a practical tool for policy makers, funders, and researchers to assess programs for investment and scaling to maximize their health impact.

## Methods

The Toronto Health Organization Performance Evaluation (T-HOPE) framework was developed using an iterative, qualitative process. Our aim was to develop a set of performance dimensions that balance what is theoretically desirable and what is empirically viable, with an emphasis on identifying measures that are credible, feasible, and comparable across health programs.

### Literature - credibility and comparability

We began by consolidating eleven existing performance frameworks on health service evaluation [14–18], social impact investment [19–21], and business process innovation [6, 22, 23]. This yielded an initial composite framework of twelve performance dimensions. In this process, we identified credible dimensions vetted by scholars and practitioners that were relevant for comparing a variety of health and business models. In consolidating frameworks from different disciplines, we focused on selecting robust dimensions applicable to a broad range of programs.

### Practice - feasibility

We next considered the performance measures that health programs are already reporting. We reviewed performance data reported by a purposive sample of 80 diverse, data-rich programs from the Center for Health Market Innovations (CHMI) database (healthmarketinnovations.org). This database catalogs over 1400 innovative health programs in LMICs, with an emphasis on private sector delivery (this includes for-profit, not-for profit, and public-private partnership (PPP) initiatives that serve poor populations in LMICs), and displays reporting provided by the programs**.** We determined the 80 programs for the sample by focusing on programs with available data in four important areas of health activity: the established fields of maternal, newborn and child health (MNCH), general primary care, and infectious diseases, plus the emerging area of mHealth. We supplemented the data available on these 80 programs in the CHMI database by collecting data from publically available sources through an online search of program websites and reports, journal articles, and news websites.

In our review of these 80 programs, our aim was to determine the types of measures programs are already reporting, to assess feasibility, while maintaining comparability by identifying common measures reported by a range of programs from different health areas with different models. The assessment included programs operating in diverse health areas, such as MNCH, eye care, tuberculosis, primary care, family planning and reproductive health. The programs commonly employ innovative operational models, such as social franchising, public private partnerships, clinic chains and networks, mobile clinics, social marketing, microinsurance, and use of mobile health technologies. Through this review, we identified performance dimensions in our initial composite framework that a variety of innovative health programs also are reporting data on, updating our framework to reflect this aspect of feasibility.

We then refined our initial framework by reviewing the relevant literature on each of the performance dimensions, including academic publications and technical reports. This review sought to strengthen the definitions and measurement approaches in a way that provides a relevant balance of our three desired characteristics:

- *Credibility*: Consistent with ideas commonly presented in the literature
- *Feasibility:* Based on existing reporting, requiring limited time and effort to provide data
- *Comparability*: Programs engaging in different health areas and models could report on the dimension

## Results and discussion

Through this process, we developed the T-HOPE framework, which includes three categories of performance – health status, health access, and operations/delivery. Within the three categories, there are fourteen subcategories of performance: three fields with definitions for health status, three for health access, and eight for operations/delivery. Table 1 summarizes the framework, providing definitions, indictors, and examples of each dimension. We also drew from the literature to identify seven descriptive fields, which Table 2 summarizes. The descriptive fields are useful for building profiles and understanding the context of specific programs.

**Table 1:** T-HOPE framework: performance dimensions

Performance dimension	Example indicators	Examples from CHMI profiles: healthmarketinnovations.org
A. Health Status		
A1. Population Coverage: Volume of clients served as a percentage of a defined target population per reporting period.	• Percentage of the target population using program services or products per reporting period.	Bangladesh Urban Primary Health Care Project (Bangladesh): Between 1998 and 2011, the primary care program had covered 82.6 % (approximately 7.78 million) of the target population in Bangladesh.
A2. Health Output: Quantitative evidence about the number of health services/products provided and/or clients served/trained per reporting period.	• Number of clients served per reporting period. • Number of products provided per reporting period.	World Health Partners (WHP) (India, Kenya): 25,836 patient visits conducted by WHP providers between January 1, 2013 and December 31, 2013.
A3. Health Outcome: Quantitative evidence of impact on intermediate or long-term health outcomes demonstrated by changes in learning, actions, and/or health status of clients/target population per reporting period.	• Change in mortality rate in target population per reporting period. • Change in disease incidence in target population per reporting period. • Change in uptake of healthy behaviours in target population per reporting period.	Deepak Foundation Gujarat (India): Initiated in 2005 to save lives and promote institutional deliveries, the Foundation’s Safe Motherhood and Child Survival Project observed a 38.7 % decline in maternal mortality from 430 cases per 100,000 live births in 2005 to 263 in 2011.
B. Health Access		
B1. Affordability: Quantitative evidence about the price of services and products compared to the average price of similar services and products in the local context, or as a proportion of income at a given time period.	• Price of service/product compared to price of the same service of a local competitor at a given period. • Price of product/service as a proportion of average household income at a given period. • Product/service provided for free.	PROSALUD (Bolivia): Charges approximately US$4 for an appointment with a general practitioner, compared to US$28 in the private sector.
B2. Availability: Quantitative evidence about the ability of clients/patients to access health services at the needed place and time per reporting period.	• Number of providers, facilities or hospital beds per target population per reporting period. • Average geographic distance or time needed for the target population to reach the facility. • Percentage of health care workers absent from the facility per reporting period. • Change in stockouts of medications or supplies per reporting period. • Hours of facility operation per reporting period.	Hygeia Community Health Plan (Nigeria): Hygeia has achieved a 95 % reduction in stock outs of anti-malarials and other essential drugs among its network of providers between January 2007 and December 2010.
B3. Pro-Poor Targeting: Proportion of clients that are economically disadvantaged and criteria used to identify and target the poor; includes whether the program is targeting a poor area or targeting the most disadvantaged group within a population.	• Percentage of a program’s clients that are in the bottom 20 % income quintile. • Percentage of a program’s clients living on less that US$2 per day. • Percentage of a program’s clients that live in a predominantly poor area.	HealthyBaby/HealthyLife Vouchers (Uganda): A 2010-2011 survey found that 29.3 % of women using the HealthyBaby voucher were in the poorest quintile of the household wealth index.
C. Operations/Delivery		
C1. Clinical Quality: Quantitative evidence of providing safe, evidence-based care, which can include comparison to other providers of similar services, and/or demonstrating change over time.	• Medical error rate per time period. • Surgical complication rate compared to providers of similar services. • Percentage of cases meeting predetermined quality standards. • Percentage of patients receiving appropriate care according to approved guidelines. • Readmission rate per time period.	Aravind Eye Care System (India): Aravind has managed to keep its infection rates low, with an average of about 4 cases per 10,000 patients, compared to an average of 6 per 10,000 in the U.K.
C2. User Satisfaction: Quantitative or qualitative evidence that is collected using a systematic methodology and reflects the clients’ perceptions of the quality of services provided.	• Client renewal rates. • Client retention rates. • Percentage of patients satisfied with services based on patient survey. • Percentage of patients that would recommend the program to others.	Red Segura Nicaragua (Nicaragua): In a customer satisfaction survey conducted in 2011, the average score women of reproductive age gave to the medical attention they received at Red Segura clinics was 4.8 on a scale of 1 to 5, with 5 being the highest quality of care.
C3. Management Quality: The procedures, systems, and processes the program has implemented to strengthen quality in key aspects of operations and delivery.	• Description of implementing a monitoring and evaluation system. • Description of establishing a Board of Governors to provide guidance and oversight. • Description of internal audit conducted on a regular basis. • Description of accreditation or certification by a reputable organization. • Description of receiving international awards for excellence and/or achievement.	Mahila Swahsta Sewa (Nepal): Quality assurance mechanisms include: 1) Quality assurance visits focused on the service delivery of intrauterine devices (IUDs) using the Lot Quality Assurance Sampling Method; 2) Development and use of quality monitoring checklists; 3) Quality action plans to address issues.
C4. Economic Efficiency: Quantitative evidence about the cost of delivering the product/service to patients/clients.	• Unit cost of providing a service/product for a single client/patient. • Average total cost to provide services/products to clients/patients.	Operation ASHA (Cambodia, India): Operation ASHA has developed a model in which the cost of providing complete TB treatment to a patient is US$80, as compared with the cost of US$300 among other not-for-profit organizations.
C5. Non-Economic Efficiency: Quantitative evidence about how long it takes for a program to deliver a product/service compared to a previous reporting period or providers of similar services.	• Patient or procedure volume per time period compared to a previous reporting period. • Patient or procedure volume per time period compared to providers of similar products/services.	RapidSMS Malawi (Malawi): The RapidSMS mHealth data collection system results in a significant reduction in data transmission delay. While Malawi’s current paper-based system takes 1–3 months to transmit child nutrition data, the RapidSMS system takes only 2 minutes.
C6. Human Resources Supply: Description of the program’s human resources supply and strategy to recruit, retain, and train staff.	• Description of initiatives that seek to promote recruitment or retention of staff. • Description of staff training programs. • Turnover or retention rate per reporting period. • Description of staff satisfaction and/or factors contributing or detracting from recruitment and retention.	Living Goods (Kenya, Uganda): Community health promoters are trained to provide basic health counselling on a variety of topics to their communities and make a modest living by selling health products. All health promoters are trained to give basic public health counselling on the use of products and to facilitate referrals to acutely ill patients. Field agents meet community health promoters at least once a month to resupply, collect payments, communicate current promotions, and provide ongoing health education and business coaching.
C7. Political Support: Qualitative evidence of a relationship or partnership with a local, regional, or national government entity.	• Description of financial or technical support from a local, regional, or national government entity. • Description of authorization of activities by a government entity. • Description of successful advocacy resulting in policy change. • Description of providing training for government officials.	Chiranjeevi Yojana (India): This program aims to reduce maternal and infant mortality through government contracts with private providers. Qualified providers sign a memorandum of understanding with the district government and are financially compensated for deliveries provided to eligible patients.
C8. Financial Management: Financial data related to the program’s balance sheet, income statement, cash flows, and ratios, concepts and calculations.	• Value of total assets at the end of the reporting period. • Net income resulting from all business activities during the reporting period. • The net cash flow of the organization during the reporting period, which is calculated by subtracting outflows from inflows of cash and cash equivalents. • Value of equity and/or other financial contributions in the organization provided by the entrepreneur(s) at the time of investment.	Naya Jeevan (Pakistan): The operational revenue of this microinsurance program in Pakistan increased by 350 % between 2010 and 2011; earned income increased from US$2850 in 2010 to US$10,500 in 2011.

**Table 2:** T-HOPE framework: descriptive fields with examples

A. Program Profile:
Description of several operational aspects of the program.
Example: Marie Stopes International (MSI) Bolivia.
1. Summary (100 word overview): MSI Bolivia approaches the subjects of sexual and reproductive health in men and women through the provision of established and mobile services, aiding in the financing of services for low-income families, social marketing, and participative, inclusive education.
2. Implementing organization: Marie Stopes International.
3. Health focus: Family Planning & Reproductive Health.
4. Type of product/service: Reproductive service delivery at medical centers and mobile clinics; a call center to provide info and make referrals; social marketing about new contraceptive products; and education in regards to sexual and reproductive rights, as well as sexual violence.
5. Client type: Young adults (13–24); men; women; general population.
6. Program type: Country office of an international organization.
7. Country registered and legal status: Bolivia, private (not-for-profit).
8. Country of Operation: Bolivia.
9. Geographic location (within the country): La Paz, Santa Cruz, Cochabamba, Potosi, Chuquisaca, Oruro, Beni. Operates in 4 of 9 states, and reaches more than 110 municipalities with 5 mobile units.
10. Stage: Existing/expansion stage.
11. Year launched: 1995.
12. Number of facilities: 11 (6 established health centers, 5 mobile clinics).
13. Number of employees: 50–99; MSI Bolivia manages a staff of 70 professionals, including doctors, drivers, health promoters and social marketing experts.
14. Target population: Approximately 800,000 people.
15. Operational and technical partners: None.
B. Problem:
Brief description of the problem that the program is trying to address, including the rationale and/or justification for the program.
Example: MotoMedics (Vietnam).
In a city like Hanoi where traffic is a critical problem, traditional ambulance vans struggle to reach patients within 30 or 45 minutes after the call for assistance is made. By then, the chance to provide life-saving medication or procedures significantly decreases. The introduction of a medical first responder program using motorbikes would improve response times and could significantly increase medial emergency survival rates as well as lower the costs of medical care for the patient.
C. Goal:
Description of the program’s theory of change or what the program aims to achieve through its interventions.
Example: Ziqitza - Dial 1298 for Ambulance (India)
Dial 1298 for Ambulance, delivered by Ziqitza Health Care Limited (ZHL), strives to deliver a nationwide network of Life Support Ambulance Services accessible to anyone, anytime and anywhere through an easy to remember four-digit telephone number. The program is committed to meeting international quality standards in emergency medical services and aims to extend the availability of emergency transportation and care to lower-income populations.
D. Process:
Description of how the program achieves its goals. This field should outline the processes and steps that are used to deliver the program’s products and/or services and the relationships between them.
Example: Piramal E-Swasthya (India).
1. Local literate women are recruited to undergo a rigorous training program in which they are trained to collect simple diagnostic information, and to provide preventive medicine, first-aid and customer service.
2. These women are given a medical kit, marketing material and a mobile phone. They are then assisted in setting up a tele-clinic (Piramal e-Swasthya Center) at their own homes.
3. Villagers who feel ill come to the Piramal e-Swasthya Center or are given a home visit. After talking to and examining the patient, the health care worker communicates this diagnostic data through a cell-phone to a centralized call center.
4. A call center paramedic enters the information provided into a simple e-diagnosis system, which generates an automated response with the recommended prescription and treatment. Doctors manning the call center also validate this.
5. If the ailment appears serious, the call center recommends that the patient visit a secondary or tertiary health care facility immediately.
6. The health care worker also conducts preventive health workshops, which generate awareness about issues such as sanitation, nutrition, and first aid.
E. Challenges/Opportunities:
Description of the obstacles the program faces in delivering its products or services, and/or any opportunities the program has discovered and plans to leverage.
Example: Aceh Besar Midwives with Mobile Phones (Indonesia).
This 2006 World Vision project leveraged mobile phone communication technology in Indonesia by distributing cell phones and developing an SMS data collection system, which helped to facilitate patient data collection by midwives and voice communication between midwives and obstetrician-gynecologists. Challenges faced in the use of these technologies included high cost of adoption, inadequate health care facilities, and poor infrastructural support.
F. Strategic Planning:
Description of how the program sets its plans for identifying and achieving future goals including scaling-up or plans for growth. This section should include plans for engaging in activities to obtain resources and assigning responsibilities to attain these goals. This section should also provide information on the future plans of the program.
Example: LifeNet International (Burundi, East Africa).
Through their efficient social franchising model, which involves medical training, management training, pharmaceutical supply, and growth financing for existing clinics, LifeNet plans to double the quality of care received in 10 million patient visits to 1,000 partner clinics in 10 East African countries by 2020.
G. Innovative Practices:
Description of innovative practices used by the program to meet its goals.
Example: APOPO (Tanzania and Mozambique).
Using process reengineering, APOPO trains African giant pouched rats in Tanzania and Mozambique to provide second-line screening of TB samples from collaborating TB diagnostic centers.

All examples are taken from CHMI program profiles (healthmarketinnovations.org)

Table 3 reports the frequency of reporting for each performance dimension by the 80 CHMI programs in our sample (i.e., the proportion of the 80 programs that report data for each framework dimension). The table also disaggregates the frequency of reporting based on subgroups for health area, type of innovation, and legal status. While there is substantial variation across subgroups, a large majority fall within the 50 % range around the mean reporting frequency value for each of the 14 performance dimensions.

**Table 3:** Frequency of reporting by 80 CHMI programs for 14 performance dimensions (% reporting), including subgroups based on health area, innovation, and legal status

Performance dimension	Total (% reporting)	Subgroup: health area				Subgroup: innovation			Subgroup: legal status		
		MNCH	Primary care	Family planning and reproductive health	Infectious disease (malaria, TB, HIV/AIDS)	Financial	Provider training	mHealth	Not-for-profit	For-profit	PPP
A. Health Status											
A1. Population Coverage	13 %	14 %	9 %	12 %	19 %	18 %	16 %	11 %	17 %	0 %	11 %
A2. Health Output	88 %	77 %	92 %	92 %	89 %	100 %	84 %	86 %	91 %	94 %	72 %
A3. Health Outcome	45 %	64 %	28 %	65 %	56 %	49 %	46 %	40 %	59 %	13 %	39 %
B. Health Access											
B1. Affordability	54 %	55 %	56 %	58 %	44 %	67 %	57 %	60 %	63 %	63 %	22 %
B1. Availability	30 %	23 %	31 %	38 %	33 %	33 %	41 %	31 %	30 %	38 %	22 %
B3. Pro-Poor Targeting	23 %	41 %	28 %	31 %	22 %	36 %	19 %	17 %	26 %	25 %	11 %
C. Operations/Delivery											
C1. Clinical Quality	26 %	45 %	9 %	42 %	41 %	36 %	30 %	17 %	33 %	19 %	17 %
C2. User Satisfaction	33 %	23 %	38 %	35 %	33 %	38 %	30 %	31 %	39 %	31 %	17 %
C3. Management Quality	30 %	45 %	41 %	42 %	26 %	44 %	30 %	29 %	30 %	31 %	28 %
C4. Economic Efficiency	21 %	14 %	19 %	27 %	30 %	28 %	22 %	20 %	26 %	13 %	17 %
C5. Non-Economic Efficiency	15 %	9 %	6 %	8 %	7 %	18 %	19 %	17 %	15 %	25 %	6 %
C6. Human Resources Supply	58 %	73 %	41 %	77 %	70 %	62 %	100 %	57 %	74 %	25 %	44 %
C7. Political Support	40 %	41 %	34 %	50 %	44 %	49 %	41 %	37 %	39 %	6 %	72 %
C8. Financial Management	84 %	82 %	84 %	81 %	74 %	87 %	84 %	91 %	80 %	94 %	83 %
Summary statistics											
Cases (a)		22	32	26	27	39	37	35	46	16	18
Minimum	13 %	14 %	6 %	8 %	7 %	18 %	16 %	11 %	17 %	0 %	6 %
Maximum	88 %	82 %	92 %	92 %	89 %	100 %	100 %	91 %	91 %	94 %	83 %
Mean	40 %	44 %	37 %	47 %	42 %	47 %	44 %	39 %	45 %	34 %	33 %
No. in bottom 25 % (b)		0	2	0	1	0	0	0	0	4	2
No. in top 25 % (b)		2	0	1	1	1	1	0	0	1	1

(a) Numbers within the “health area” and “innovation” subgroups sum to more than 80 cases because some programs engage in multiple activities

(b) “No. in bottom (top) 25 %” indicates number of cases in the 14 performance dimensions in each column that are less than half (more than 1.5 times) the mean percentage in the performance dimension

Implications: Despite substantial variance, most subgroups provide similar frequency coverage, almost all falling within the 50 % range around the mean frequency value for each category. The “for profit” legal status subgroup is the most likely to fall below the 50 % coverage range (4 of 14 categories); no other subgroup has more than two categories that fall below the 50 % coverage range; for-profit programs may have lower reporting rates due to weaker incentives to disclose data that is not considered relevant to their bottom line

This framework can be used to understand a program’s performance, including its activities, goals, and organizational context. The dimensions are framed and defined in a manner that balances comprehensiveness with comparability across diverse programs. By systematically applying the criteria in the framework, diverse stakeholders including program managers, funders, and researchers may achieve an understanding of relative program performance.

### Illustrative comparisons

To illustrate the framework, Tables 4, 5 and 6 compare ten programs, two providing eye care services, five in mHealth, and three in MNCH. Together, the ten cases provide comparison for all fourteen categories in the T-HOPE framework. We summarize the comparisons here, in terms of their implications for funders, researchers, and program managers.

**Table 4:** T-HOPE framework: comparision of eye care programs (two programs)

	Comparative features	Program Eye Care 1 (Latin America)	Program Eye Care 2 (South Asia)
Overview		Program Eye Care 1 is a for-profit program that provides eye care services and specializes in cataract surgeries using a high-volume, low-cost approach. It operates several vision centers, a surgical hub, and provides outreach activities in the national capital.	Program Eye Care 2 is a not-for-profit rural hospital that focuses on performing high-volume, low-cost eye surgeries in the country. The hospital is located in one major city, with a satellite facility in a second city.
Population Coverage (A1)	Program Eye Care 2 has 80 % market share for its operations compared to 2.5 % for Program Eye Care 1.	In 2012, the program’s market share was estimated at 2.5 % in the urban part of the country.	In 2011, the program had approximately 80 % of the market share within its catchment area.
Health Output (A2)	Program Eye Care 2 provides more than 30 times as many eye surgeries a year as Program Eye Care 1.	5,400 cataract surgeries were performed from 2010–2012.	95,243 surgeries were performed from 2010–2011.
Affordability (B1)	Both programs are providing eye surgeries that are much lower in cost and performed more efficiently than their private and public competitors. Indeed, both programs provide surgeries that are approximately half the cost of similar services in the local context.	As of 2013, cataract surgeries cost approximately US$465, half the national average of US$1240.	The cost of cataract surgery is US$33 for a middle class patient compared to around US$50–US$60 in bigger cities.
Pro-Poor Targeting (B3)	Both programs focus on serving the poor, although a higher proportion of Program Eye Care 1’s patients are from an economically disadvantaged group.	85 % of patients treated are living at the bottom of the pyramid.	The program focuses on serving impoverished, rural communities. Over 50 % of the services it delivers are free or subsidized for poor patients.
Clinical Quality (C1)	Both have surpassed the WHO’s recommended guidelines for visual acuity after cataract surgery, suggesting quality is high.	While 53 % of patients had visual acuity less than 20/200 before surgery, 87 % ended up with best corrected visual acuity (BCVA) greater than 20/60 (equivalent to the WHO benchmark of 6/18).	From 2007–2008, 81 % of patients receiving small incision cataract surgery operations had BCVA <3/60 (blinding cataract) before surgery; BCVA at 6 weeks after operation was ≥6/18 in 87 % of operated eyes. (The WHO recommends that after cataract surgery, at least 85 % of operated eyes should have visual acuity ≥6/18 and less than 5 % of operated eyes should have BCVA <6/60) [33].
Non-Economic Efficiency (C5)	Program Eye Care 2’s surgeons are able to perform 100 times as many surgeries as their local competitors, while Program Eye Care 1 performs 10 times as many as their local competitors. While these differences seem substantial, with the comparison in Program Eye Care 2’s favor, it should be noted that Program Eye Care 2 is more established than Program Eye Care 1, having launched 10 years earlier.	Program surgeons perform 100 cataract operations per month, compared to an average of 7–10 per month conducted in private hospital settings.	On average, 250–300 cataract surgeries are performed per day, compared to 3–5 surgeries a day performed by the nearby government hospital. Due to its innovative operational practices, its surgeons can perform a cataract surgery in one third of the industry standard time.

The text summarizes the implications of these comparisons

**Table 5:** T-HOPE framework: comparison of programs using mHealth (five programs)

	Comparative Features	Program mHealth 1 (South Asia)	Program mHealth 2 (South Asia)	Program mHealth 3 (SubSaharan Africa)	Program mHealth 4 (South America)	Program mHealth 5 (South Asia)
Overview		A for-profit hospital using management software and a high- volume, low-cost approach to provide heart surgeries.	A not-for-profit program using a telemedicine call center and community health workers to provide primary care services.	A not-for-profit program where community health workers collect children’s health data on mobile phones, with monitoring by a primary care doctor.	A not-for-profit program that provides reproductive service delivery at medical centers and a call center.	A PPP with a charitable organization operating government primary health centers, some of which provide telemedicine services.
Health Output (A2)	Program mHealth 1, 2, 4, and 5 serve several thousands of patients a year, while Program mHealth 3 is more focused.	From 2001 to 2007, the program performed over 23,000 surgeries and 34,000 catheterization procedures.	From 2008 to 2011, the program treated 40,000 patients in 200 villages.	From 2009 to 2012, over 1400 children were enrolled in the program by their parents; 900 children are actively being served.	In 2010, the program had 15,000 monthly average clients, providing consultations, lab services, vasectomy, tubal ligation, IUDs, injectables, implants, pills, condoms, and emergency contraception.	In 2012, the program reached 1 million people through its primary health centers.
Health Outcome (A3)	Programs mHealth 3, 4, and 5 report strong levels and gains in health outcomes that merit study on how the programs and/or other sources achieved them.			Behaviour change and improved access increased the rate at which subscribers visit health facilities; a subscriber to the program visits the health care center at least 3 times per year on average, whereas the average user rate in the district is 1.05.	In 2010, with 71,454 CYPs generated, it achieved a 31 % increase in CYP’s over the previous year.	From 1996 to 2007, in states served by the organization’s primary health care centers: Infant mortality dropped from 75 % to 24 %; still birth from 38 % to 10 %; perinatal mortality from 68 % to 17 %; neonatal mortality from 70 % to 10 %; child mortality (1 ~ 5 years) from 12 % to 3 %; under-5 mortality from 88 % to 27 %.
Affordability (B1)	All five programs offer more affordable services than other options available locally.	In 2012, the program charged US$2,400 for heart surgery, compared to US$5,500 charged at an average private hospital in the country.	In 2012, the program provided free consultations.	In 2012, families paid a monthly subscription fee of about US$1 for the package of services per child. This is the equivalent of a kilo of onions, a price affordable to low-income families in the urban areas.	In 2012, the cost of a medical consultancy in facilities is US$4.30 compared to US$10 in the local market.	All services at primary health centers are provided free of cost.
Availability (B2)	Programs mHealth 3, 4, and 5 provide models for gains in availability of health care services.			In 2012, the program improved access to immediate health care for 20 % of the local families by an average of 15 km; travel time was reduced by 4 hours or more.	In 2012, the call center operated Monday to Friday, 8:30 am to 7 pm, and Saturday, 9 am to 1 pm. The call center has a national number and can be called by individuals anywhere in the country.	In 2012, all primary health centers operated 24 hours a day, 7 days a week.
Pro-poor targeting (B3)	Program mHealth 3 has a particularly high proportion of poor patients, while program mHealth 1 provides subsidies to a meaningful share of its patients.	The program has subsidized poor patients to the tune of US$2.5 million, which benefited close to half of the patients that came to the program for treatment.		90 % of the program’s subscribers report having unstable earnings.		
User Satisfaction (C2)	Programs mHealth 2 and 3 have high patient satisfaction rates, with program mHealth 3 having a slightly higher rating. Program mHealth 4 provides an example of how to increase patient ease.		The program has received an 85 % patient satisfaction rating consistently over the last year from patient feedback surveys.	A 2009 evaluation survey carried out by a PhD student under the supervision of a national agency showed that 96 % of the enrolled families are satisfied with the service.	Market research found that clients were intimidated by white-coated doctors and sterile environments, which they associated with illness rather than health. With trained staff performing most consultations and providing advice in friendlier environments, clients report feeling more at ease.	
Management Quality (C3)	Programs mHealth 2, 3, 4, and 5 offer examples of activities that can strengthen management, operations, and delivery.		The program is an ISO 9001- 2008 certified company.	The program uses a qualitative health monitoring system to ensure both low and higher income populations are served.	The program uses a standardized assessment tool for all regional programs. The evaluations improve technical and financial performance, while creating transparency and accountability.	The program uses a hospital management information system developed by a major university to improve hygiene and good maintenance.
Economic Efficiency (C4)	Programs mHealth 1, 3, and 4 offer models of achieving different aspects of financial efficiency.	The program brought down the cost of electrocardiogram machines from US$750 to less than US$300.			The operational cost to provide call center services is US$0.21/min per call to the call center, which allows the nonprofit to provide affordable services.	The operating cost of each primary health center is about US$50,000, lower than comparable facilities.
Non-Economic Efficiency (C5)	Programs mHealth 1 and 2 serve more patients in a day than other local options, while program mHealth 4 provides faster service than other local options.	The program performs 32 heart surgeries a day, about 8 times more surgeries per day than the average for other comparable hospitals.	In traditional models, a doctor could treat up to a 100 patients per day. The program’s model allows each doctor to diagnose over 400 patients per day spread across 100 villages.		The program’s tubal ligation procedure takes 20 minutes compared to 2 hours observed at other facilities.	
Human Resources Supply (C6)	Programs mHealth 2, 3, 4, and 5 provide models of training for health workers.		The program has trained over 200 local village women to become health workers.	The program started offering training sessions in 2011 for its teams as well as medical teams in the partnering health center.	The program offers ongoing training to staff to assure quality of care.	The medical officer, staff nurse, pharmacist and laboratory technician are required to stay in the same town/village where the primary health care center is located. Auxiliary nurse/mid-wife are trained to do pap smears.
Political Support (C7)	All programs partner with governments actors, using multiple models to gather support and gain contact with clients.	The program developed micro-insurance schemes with state governments, which work on flexible payments, and have helped thousands coming from low-income groups to procure services.	The program partners with the state government.	The program organized an informational event for the surrounding population in front of the District Chief’s home. Counsellors presented to the District Chief, and then the program’s employees followed suit with an information session. The program also partners with the Ministry of Health.	The program has negotiated agreements with the Ministry of Health and with local governmental units that enable them to provide services at municipal health centers. The municipality schedules visits from program staff, organizes clients, and provides places for services.	The program operates as a PPP, with the charitable organization managing government primary health centers in several states.
Financial Management (C8)	The programs offer models to learn about varied mixes of fee, donor, and government sources of revenues	Over 50 % of revenue came from heart surgeries, while 9 % came from coronary care charges and 8 % of from outpatient fees. In the financial year that ended in March 2005, the hospital earned 20 % operating profits before interest, depreciation, and taxes.		The program receives 50 % of its operating costs from subscription fees, while the other 50 % is sought from donors. The program reports that it has not yet found a sustainable economic model.	The program works with an annual revenue of about US$1.5 million; of this, 50 % is raised from fee revenue from clinic services.	90 % of operating costs are covered by state governments; the charitable organization covers 10 % of costs through donations from individual donors.

The text summarizes the implications of these comparisons

**Table 6:** T-HOPE framework: comparison of programs using MNCH (three programs)

	Comparative Features	Program MNCH 1 (South Asia)	Program MNCH 2 (South East Asia)	Program MNCH 3 (South Asia)
Overview		A for-profit hospital chain providing health care to women and children.	A not-for-profit network of franchised clinics providing maternal and child health services and family planning, reproductive health and HIV/AIDS services.	A not-for-profit clinic franchise offering services for maternal and child health, family planning and reproductive health, general primary care, tuberculosis and malaria.
Health Output (A2)	Programs MNCH 2 and 3 serve millions of clients a year, while Program MNCH 1 serves about 10,000 patients a year.	Since its inception in 2005 through the summer of 2007, the program served over 21,271 outpatients and 1,810 inpatients, of which 1,043 were there for deliveries. The program has become the largest chain in the region, treating more than 70,000 patients and delivering more than 7,000 healthy babies.	In 2010 alone, the program’s 629 centers, across 40 countries, provided 7 million couples with high quality health services, including: family planning; safe abortion & post-abortion care; maternal & child health care, including safe delivery and obstetrics; diagnosis & treatment of sexually transmitted infections; and HIV/AIDS prevention.	In 2010, the program served 9.5 million clients needing services for diarrhea, pneumonia, immunization, and child delivery in the hospital and at home.
Health Outcome (A3)	Both Programs MNCH 1 and 2 show improvements in health outcomes due to their interventions, with Program MNCH 1’s impact involving changes in healthy prenatal and delivery behaviours, and Program MNCH 2’s showing an impact in reproductive health.	Of all the women who deliver their second or third child at the program, over 50 % had their previous delivery at home or in an under-resourced government hospital; between 2011 and 2012, the average antenatal visits by the pregnant women increased from 2.5 to over 4.	The program provided 49,619 IUDs in 2011, which was the major contributor to its 283,571 CYPs generated during the period.	
Affordability (B1)	Program MNCH 3 provides free services for the poor, while Programs MNCH 1 and 2 provide services for less than other similar local offerings. Program MNCH 1 provides services for approximately one fifth the cost of similar services elsewhere, while Program MNCH 2 provides services for approximately one third to one sixth the cost of similar services elsewhere.	In 2012, the price of a normal delivery at the program was approximately US$40, compared to the standard US$200, and this includes all doctor and nurse visits, all medicines, and the complete stay in the hospital.	In 2012, the program had both mandatory and recommended pricing. For example, the price for an IUD is set at US$2. Competitive prices for an IUD in private clinics range from US$6.60–US$13. Deliveries by midwives range from US$33–US$77, whereas private doctor and hospital prices for midwives range from US$220–US$330.	In 2012, prices for services ranged between US$0 (for the poor) and US$0.38.
Availability (B2)	Both Programs MNCH 1 and 2 are roughly within walking distance of the communities they serve.	In 2012, families who patronized the hospital typically lived within a 5 km radius of the hospital. Strong word of mouth recommendations extended this radius up to 20 km.	In 2012, 81 % of the program’s facilities were within walking distance for community women.	
Pro-Poor Targeting (B3)	All programs serve poor clients. For Programs MNCH 2 and 3, approximately one third to one half of their clients are impoverished. Program MNCH 1 serves clients that are disadvantaged but not at the bottom of the pyramid.	The program targets customers from a key tier in the national population: not the very bottom of the pyramid, but those that are low down on the pyramid. Monthly family income of customers is as follows: 40 % earn below US$90 per month; 30 % earn between US$91 and US$130 per month; 20 % earn between US$131 and US$220 per month; and 10 % earn above US$220 per month. The poverty line in the region is US$31 per month.	In 2011, 46 % of the program’s clients were members of households whose incomes fell below the poverty line; 66 % were unemployed; and 78 % had at least 2 children.	One of the primary goals of the program is to serve poor patients and therefore all clinics have what is known as a poorest-of-the-poor fund. Clients that qualify as poor receive a card, which entitles them to receive free services. The official qualification process for the card is based on criteria used by the national public health department to identify lower socio-economic status, but if a client indicates that they are poor, they are provided with the card. The program reports that 27 % of its patients are poor.
Clinical Quality (C1)	All programs show impact in clinical quality in provision of clinical services.	Through the program’s long-standing partnership with a U.S. health care institute, its clinical quality indicators have shown significant improvement. For example, its “culture of safety” ratings increased from 35 % in January 2010 to 77 % in December 2010.	Through the program’s Quality Technical Assessment, 100 % of franchised-midwives were found adhering to service provision standards and having maintained confidence in their delivery of program services.	As of 2011, there were almost 6,000 safe deliveries per quarter. Only one woman had died while giving birth under the care of a franchised facility since the program’s inception.
User Satisfaction (C2)	Programs MNCH 2 and 3 show approximately 60–70 % of patients are satisfied with services. In addition, 98 % of Program MNCH 2’s patients expressed loyalty, suggesting high user satisfaction. Only 0.3 % of Program MNCH 1’s patients have filed complaints regarding services, also suggesting a high level of patient satisfaction.	Only 18 complaints from about 6000 users of inpatient services were received through the program’s complaint registration system between 2011 and 2012.	61 % of the program’s clients identified themselves to be ‘satisfied’ with regard to price and 68 % in regard to the feeling of comfort. In addition, 58 % expressed satisfaction equivalent to that of the evaluation's highest scale in terms of feeling security against conception. 98 % expressed loyalty to the program, which was primarily based on quality of services.	The clients are typically loyal users of the program’s services and the franchise found that that 71 % of customers are repeat users.
Management Quality (C3)	All programs conduct monitoring protocols to ensure high quality management and operations.	The flagship hospital was ISO 9001:2000 certified in 2007. Customer-focused service is embodied in the program’s protocol and approach whereby each employee is expected to be polite, attentive, and respectful to patients.	Clinical compliance audits, business systems audits, and franchisee and customer satisfaction surveys are conducted regularly through site visits at each franchisee. Team members help franchisees correct problems with entering data.	Not-for-profit organizations monitor clinical quality of the clinics and report findings and progress on resolving performance gaps to the program head office. A clinic level quality circle is in place and all clinic staff members are responsible for maintaining the quality of the services they provide. A clinical quality council reviews clinic performance indicators.
Economic Efficiency (C4)	Both Program MNCH 2 and 3 report on cost per CYP, with Program MNCH 3’s costs at less than half that of Program MNCH 2.		The cost per CYP generated has dropped to US$16 after 2 years - roughly on par with other franchises at similar stages of development.	The cost per CYP generated is about US$7.
Human Resources Supply (C6)	All programs report on their human resources situation. Program MNCH 1 describes efforts to attract doctors and employ other types of health workers to keep costs low. Program MNCH 2 describes training for franchisees, and Program MNCH 3 describes reasons for staff turnover.	Talent recruitment: doctors earn fixed salaries so they can focus on care of existing patients as opposed to the need to attract new customers. The program typically employs Auxiliary Nurse Midwives who undergo significantly less training than Graduate Nurse Midwives, reducing costs and attrition.	As part of staff training, franchisees must complete a minimum of 10 supervised IUD insertions, 5 IUD removals, and 10 pap smears.	Within the franchisors’ headquarters, 35 % of staff turnover was due to releasing staff for performance reasons, while 65 % of staff turnover was due to career advancement either for opportunities outside the country or in-country promotions.

The text summarizes the implications of these comparisons

#### Eye care service comparisons

Table 4 compares the performance dimensions for two facilities that provide cataract surgeries, including Program Eye Care 1, a for-profit program in Latin America, and Program Eye Care 2, a not-for-profit program in South Asia. Several implications arise for different types of stakeholders.

- *Funders***:** Funders can use the comparison to help determine high opportunity investments, based on the strength of the factors that a given funder believes are most relevant for its goals. In this example, a funder focused on primarily serving disadvantaged populations may choose to fund Program Eye Care 1 given that a greater proportion of its patients are poor or, instead, might provide funding to Program Eye Care 2 to help it serve a larger number of poor people, even if the proportion is smaller.
- *Researchers***:** Scholars can use the comparison to research innovation and performance, such as exploring how different aspects shape program performance, including the operating context (Latin America vs. South Asia, rural vs. urban), legal status (for-profit vs. not-for-profit), and model infrastructure (hub and spoke vs. hospital).
- *Program managers***:** Program managers, meanwhile, can use the comparison to identify opportunities to learn new skills and techniques. For instance, Program Eye Care 1 might seek to understand how Program Eye Care 2 grew its population coverage and learn from Program Eye Care 2’s efficiency in performing cataract surgeries.

#### mHealth comparisons

Table 5 compares the performance dimensions of five programs using mHealth, including Program mHealth 1, a for-profit hospital using management software in South Asia, Program mHealth 2, a not-for-profit telemedicine program in South Asia, Program mHealth 3, a not-for-profit mobile monitoring program in SubSaharan Africa, Program mHealth 4, a not-for-profit medical center and call center in South America, and Program mHealth 5, a PPP operating clinics with telemedicine services in South Asia.

- *Funders*: Funders such as investors may be particularly interested in partnering with Program mHealth 1, which has shown strong revenue and profits through its financial model, as well as strong performance in non-economic efficiency and management quality as evidenced by its ISO 9001-2008 certification. Donors may want to support the efforts of Programs mHealth 2 and 4, which have achieved substantial scale in providing affordable and efficient health services. Donors interested in helping a medically successful program that needs financial support may be drawn to Program mHealth 3. Public agencies and policy makers looking for PPP models may want to explore Program mHealth 5’s successful approach to partnership.
- *Researchers*: Researchers may be interested in exploring how Programs mHealth 1 and 2 are able to serve many more patients per day than other local options and the types of procedures that are amenable to this. They may want to study how these programs, both for-profit and not-for-profit, have been able to develop relationships with government entities to deliver their programs, and the advantages and challenges of doing so. Researchers may also want to study how Program mHealth 5 has contributed to improvements in local health outcomes.
- *Program managers*: Program managers may be interested in learning how Programs mHealth 2 and 3 are able to achieve high satisfaction ratings with patients, and how to scale up services to serve the large numbers of patients Programs mHealth 1 and 2 are able to serve. Program managers may also be interested in learning about the value proposition that Program mHealth 5 has used to gain substantial financial support from public bodies.

#### Maternal, Newborn, and child health (MNCH) comparisons

Table 6 compares the performance of three MNCH programs, including Program MNCH 1, a for-profit hospital chain serving women and children in South Asia, Program MNCH 2, a not-for-profit clinic franchise focusing on MNCH and reproductive health in South East Asia, and Program MNCH 3, a not-for-profit clinic franchise offering MNCH and general primary care services in South Asia.

- *Funders*: Funders may be particularly interested in the couple years of protection (CYPs) generated by programs and the ability for Program MNCH 3 to provide CYPs at a relatively low cost, choosing to support programs that are able to produce health outcomes most cost effectively.
- *Researchers*: Researchers may be interested in understanding how MNCH 1 has influenced the health behaviours of pregnant women. Given that Programs MNCH 2 and 3 are franchises, scholars may also want to explore how Health Outcome, Clinical Quality, User Satisfaction, and Management Quality compare with non-franchised MNCH programs.
- *Program managers*: Program managers may find the data on Human Resources Supply particularly relevant, including MNCH 1’s efforts to employ non-physician health workers to keep costs low, the types of training provided by Program MNCH 2 for its franchisees, and reasons for staff turnover in Program MNCH 3’s franchise model.

In these examples, the framework data give a snapshot of performance information about each program, and provide an entry point for funders, researchers, and program managers to conduct preliminary comparisons and identify avenues for further investigation. Applied at regular intervals, these performance dimensions can also help track program performance over time, providing a richer understanding of the program’s capabilities and potential. As well, to understand program performance, one must also have knowledge of program operations, goals, challenges, and processes that shape this performance; the descriptive fields framework offers relevant information that complements the T-HOPE performance framework.

### General implications

One of the key strengths of this framework is the integration of established approaches for measuring the performance of health programs and organizations. The wide variety of tools used today creates confusion, puts an inappropriate burden on delivery organizations, and fails to achieve comparability. Delivery organizations in LMICs with limited resources often have difficulty meeting the monitoring and evaluation demands placed on them by different donors, suggesting the need for greater coordination on reporting requirements and simplified measures [24, 25]. By harmonizing measurement requirements, funders may implement more effective pan-organizational strategies for achieving targeted health outcomes while reducing the reporting burden on the organizations they fund [26].

This framework can be used to highlight and compare the performance of innovative health programs for various stakeholders. However, while providing a snapshot of program performance at a moment in time, it will be of greatest value when combined with descriptive information about program activities, goals, and context that shapes this performance. It can provide an even richer understanding of program performance if applied over time to track progress. Also, while the framework can facilitate comparison of performance amongst programs and over time, given the diversity of innovative models emerging, we have not included benchmarks for the example indicators of our performance dimensions. Benchmarks will vary by health area and operational model, and program managers and others can identify whether their programs are meeting accepted standards.

While we have endeavored to develop credible, feasible, and comparable performance measures, some of the framework criteria are structurally more difficult to measure than others, as Table 3 highlights. For example, Population Coverage requires an accurate, quantified measure of a program’s target population, which may not be readily available in resource-limited settings without birth registration and accurate census information. Measuring Pro-Poor Targeting may involve complex and multidimensional considerations for identifying poor patients [27]. Assessing Health Outcome, meanwhile, may be challenging and time consuming, involving tracking patient health status after the intervention [13]; this may involve impact evaluations, requiring advance planning, additional funding, and rigorous research designs to ensure the results are attributable to the program, a research approach relatively few social development programs have been able to carry out [28].

We have included these performance dimensions in the framework because they are considered critical for assessing impact in the literature [29–31]. We have aimed to provide simple and straightforward definitions and example indicators based on the reporting of programs in the CHMI database. However, some dimensions may require additional information and knowledge that is not as easily accessible for new and small-scale programs as it is for large-scale, established ones. Greater technical and financial support is needed from stakeholders such as funders and researchers to assist program managers with reporting on this valuable data [7, 28]. In addition, further field-testing of the framework can help to refine these performance dimensions so they are more attainable for program managers and also help to identify more feasible methods for program managers to access this information in resource-constrained contexts.

Despite these limitations, the development of an integrative framework that acknowledges and balances the tradeoffs between credibility, feasibility, and comparability is urgently needed. This could benefit programs interested in understanding and communicating their activities and accomplishments; funders making decisions on which programs to support; and researchers seeking to better understand performance of innovative health care delivery models and programs. This framework also aims to encourage greater discussion on the types of metrics needed to meaningfully and cost-effectively understand program performance, identifying areas for improvement and opportunities for further collaboration and discourse amongst different groups with shared interests in global health.

## Conclusions

The T-HOPE framework is designed to cultivate the adoption of performance measures that meet the needs of diverse programs, while encouraging collaboration, coordination, and sharing of knowledge among programs, funders, and researchers. In doing so, the framework provides an important step towards accurately and realistically assessing the health impact and sustainability of programs aiming to meet the needs of the poor.

In practice, this framework has been incorporated into CHMI’s Reported Results initiative [22]. Through this initiative, programs can display public profiles with reporting on selected performance dimensions. The T-HOPE approach has also informed the Impact Reporting and Investment Standards’ (IRIS) [32] health working group of the Global Impact Investment Network in the development of a core set of health metrics for social enterprises. The resulting IRIS metrics, while focused on a small number of process measures that are pertinent to clinics and hospitals, have been selected to enhance comparability. In parallel, the more comprehensive T-HOPE framework allows for comparisons across a wider range of program types, and may be used to describe tradeoffs between quality, cost, and accessibility. Thus, the approaches are complementary: IRIS metrics may be used to scan for promising activities among hospitals and clinics, while the T-HOPE framework can be used to structure in-depth analyses and comparison of health programs.

The collection of credible, feasible, and comparable information on health organization performance is essential for identifying effective and innovative approaches to delivery. By understanding and comparing the performance of health programs, we can better determine which models are generating innovations that create health impact and real value in LMICs. Such understanding is crucial to progress.

## Abbreviations

BCVA: Best corrected visual acuity
CHMI: Center for Health Market Innovations
CYP: Couple years of protection
IRIS: Impact Reporting and Investment Standards
IUD: Intrauterine device
LMIC: Low- and middle-income country
MNCH: Maternal, Newborn, and Child Health
PPP: Public-Private Partnership
T-HOPE: Toronto Health Organization Performance Evaluation
